# Direct Sensing of Nutrients via a LAT1-like Transporter in *Drosophila* Insulin-Producing Cells

**DOI:** 10.1016/j.celrep.2016.08.093

**Published:** 2016-09-27

**Authors:** Gérard Manière, Anna B. Ziegler, Flore Geillon, David E. Featherstone, Yael Grosjean

**Affiliations:** 1 CNRS, UMR6265 Centre des Sciences du Goût et de l’Alimentation, 21000 Dijon, France; 2 INRA, UMR1324 Centre des Sciences du Goût et de l’Alimentation, 21000 Dijon, France; 3 Université de Bourgogne Franche-Comté, UMR Centre des Sciences du Goût et de l’Alimentation, 21000 Dijon, France; 4 Biological Sciences, University of Illinois at Chicago, Chicago, IL 60607, USA

**Keywords:** amino acid transporter, minidiscs, food, starvation, *Drosophila* insulin-like peptides, insulin-producing cells, glutamate dehydrogenase, glycemia, growth, *Drosophila*

## Abstract

Dietary leucine has been suspected to play an important role in insulin release, a hormone that controls satiety and metabolism. The mechanism by which insulin-producing cells (IPCs) sense leucine and regulate insulin secretion is still poorly understood. In *Drosophila*, insulin-like peptides (DILP2 and DILP5) are produced by brain IPCs and are released in the hemolymph after leucine ingestion. Using Ca^2+^-imaging and ex vivo cultured larval brains, we demonstrate that IPCs can directly sense extracellular leucine levels via minidiscs (MND), a leucine transporter. MND knockdown in IPCs abolished leucine-dependent changes, including loss of DILP2 and DILP5 in IPC bodies, consistent with the idea that MND is necessary for leucine-dependent DILP release. This, in turn, leads to a strong increase in hemolymph sugar levels and reduced growth. GDH knockdown in IPCs also reduced leucine-dependent DILP release, suggesting that nutrient sensing is coupled to the glutamate dehydrogenase pathway.

## Highlights

• IPCs directly sense extracellular leucine levels via minidiscs (MND)
• MND knockdown in IPCs abolishes loss of DILP2 and DILP5
• This leads to a strong increase in hemolymph sugar levels and reduces growth
• GDH knockdown in IPCs reduces leucine-dependent DILP release

## Graphical Abstract

**Figure:**
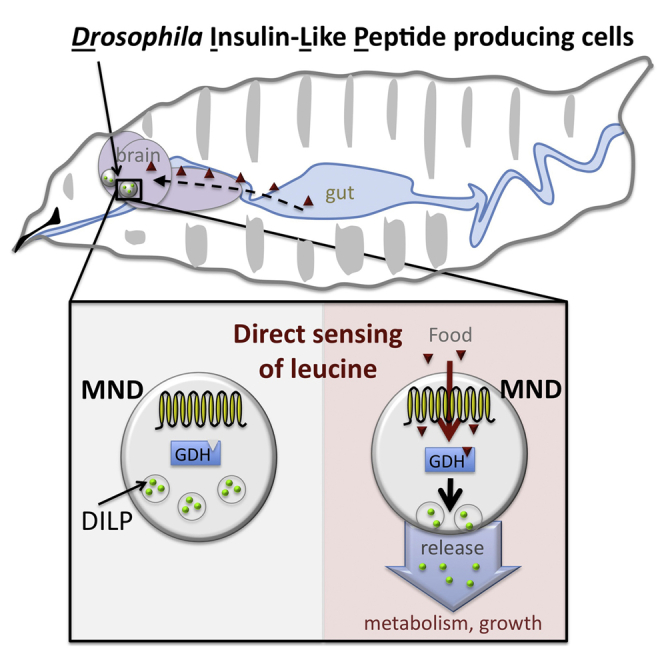

## Teaser

Manière et al. find that leucine induces the disappearance of two DILPs in *Drosophila* IPCs. Minidiscs (MND) is the primary leucine sensor, and downregulation has consequences for glycemia and growth. The authors propose that the leucine/MND pathway represents a conserved mechanism for insulin release.

## Introduction

Nutrients are essential for survival, growth, and fitness in all organisms. In response to nutrient stimuli, several hormones, such as insulin, leptin, and ghrelin, are produced to regulate energy balance (Bahary et al., 1990, Banting and Best, 2007, Kojima et al., 1999). For decades, insulin secretion from pancreatic β-cells was thought to be primarily controlled by blood sugar levels (Fu et al., 2013). Increasing evidence indicates that insulin release is also controlled by dietary amino acids (Sener et al., 1981, van Loon et al., 2003, Zhang and Li, 2013). In particular, the essential amino acid L-leucine (leucine) has proven to stimulate insulin release in cultured pancreatic β-cells (Sener et al., 1981). Recently, Cheng et al. (2016) proposed that the system-L amino acid transporter LAT1 is required for regulating cell signaling and function in β-cells.

*Drosophila melanogaster* has emerged as an excellent model organism to study the role of nutrients such as sugars, amino acids, and lipids on insulin-dependent metabolism (Géminard et al., 2009, Ikeya et al., 2002, Padmanabha and Baker, 2014). Eight *Drosophila* insulin-like peptides (DILPs) have been identified so far (Brogiolo et al., 2001, Colombani et al., 2012, Garelli et al., 2015). DILP2, DILP3, and DILP5 are mainly secreted by a bilateral cluster of insulin-producing cells (IPCs) located within the *pars intercerebralis* in the brain, the functional analogs of mammalian β-cells in pancreatic islets (Bai et al., 2012, Brogiolo et al., 2001, Rulifson et al., 2002). These three DILPs are involved in lipid storage, dietary restriction, and sugar metabolism (Bai et al., 2012, Broughton et al., 2008, Chatterjee et al., 2014, Grönke et al., 2010, Ikeya et al., 2002). It has been reported that secretion of DILP2 and DILP5 depends on feeding status (Buch et al., 2008, Géminard et al., 2009, Agrawal et al., 2016). They are stored within IPCs in starved larvae and released after feeding. Experiments by Géminard et al. (2009) suggested that specific amino acids including leucine are involved in nutrition-dependent DILP secretion.

The current model proposes that IPCs only indirectly sense nutrients such as amino acids. In this model, the primary nutrient sensor is the fat body, which is most likely the functional analog of the vertebrate adipose tissue. After feeding, the fat body secretes several hormones, which communicate to the IPCs that nutrients have arrived through feeding (Géminard et al., 2009, Koyama and Mirth, 2016, Rajan and Perrimon, 2012, Sano et al., 2015). Four of those fat body-secreted hormones have been described in *Drosophila*. The first one (UPD2) is a type-I cytokine-related protein, which seems to be a functional homolog of mammalian leptin. UPD2 is secreted by the fat body after the ingestion of a diet containing sugars or lipids. Consequently, UPD2 represses inhibitory neurons, which make contacts with IPCs to trigger DILP2 release (Rajan and Perrimon, 2012). The second hormone (CCHAMIDE-2) is a peptide produced by the fat body and by gut endocrine cells in response to a diet containing glucose or yeast, but not in response to amino acids like leucine. CCHAMIDE-2 is believed to positively stimulate the release of DILP2 and DILP5 (Sano et al., 2015). Additionally, the fat body produces and secretes two growth-blocking peptides (GBP1 and GBP2) in response to dietary amino acids. These enhance the release of DILP2 from IPCs (Koyama and Mirth, 2016).

Thus, the current model suggests that ingested nutrients regulating metabolism and food intake are only indirectly sensed, and that several hormones are compulsory to communicate nutrient status from peripheral tissues (like the fat body or the gut) to IPCs (Géminard et al., 2009, Koyama and Mirth, 2016, Rajan and Perrimon, 2012, Sano et al., 2015).

Here, we demonstrate that, in *Drosophila,* leucine induces the secretion of both DILP2 and DILP5 by IPCs in a direct way without the requirement for a hormonal signal. Using Ca^2+^-imaging and ex vivo brain cultures, we deciphered how leucine leads to the release of DILP2 and DILP5. We identified a *Drosophila* homolog of the mammalian L-type amino acid transporter 1 (LAT1), called minidiscs (MND), as the primary leucine “sensor” in IPCs. We show that IPCs require MND for leucine to induce DILP2 and DILP5 secretion, and that this process also depends on the glutamate dehydrogenase (GDH) pathway. Furthermore, the downregulation of MND leads to an increase in glycemia and causes growth defects. Taken together, our data show that direct leucine sensing via LAT1-like amino acid transporters is an evolutionarily conserved mechanism of IPCs in vertebrates and in invertebrates.

## Results

### Leucine and Isoleucine Induce Neuronal Activity in Larval IPCs

**Figure 1:**
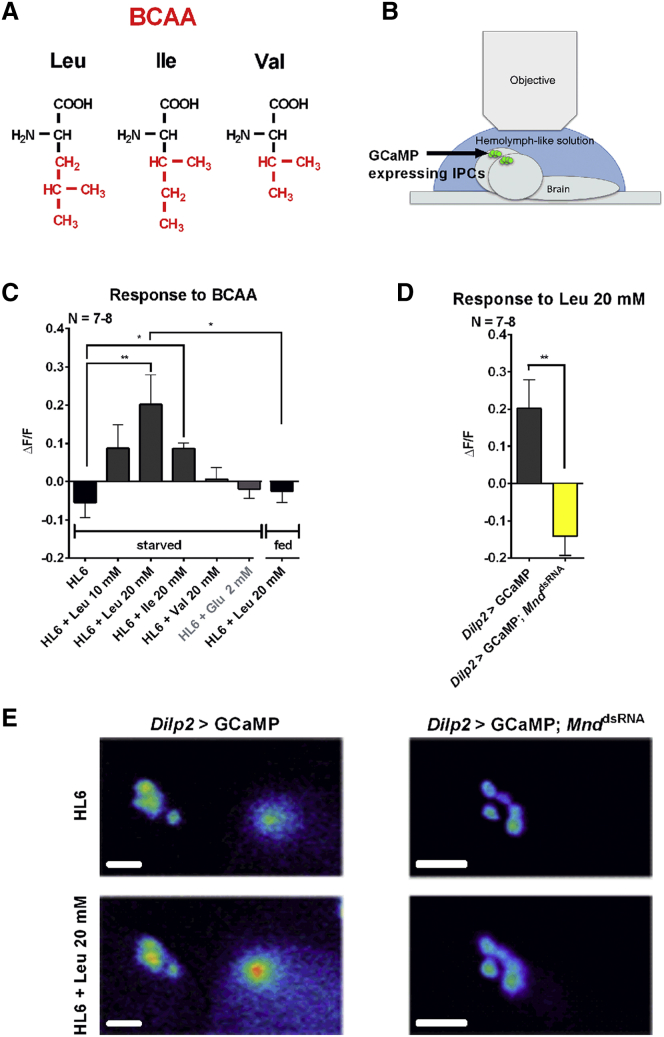
Real-Time Calcium Imaging of Larval IPCs Exposed to Proteinogenic Branched-Chain Amino Acids (A) Structure of the proteinogenic BCAAs. (B) The calcium sensor GCaMP3 is expressed in brain IPCs (green) and reflects their neuronal activity. The brains of third-instar larvae are exposed to a HL6 in which BCAAs are directly added. (C) Changes of IPC neuronal activity of a control genotype (*Dilp2*-Gal4 > UAS-*GCaMP3*) to various BCAAs and a non-BCAA as a control (glutamate) are reflected by a change in GCaMP3 fluorescence. The animals were starved for 24 hr prior to the experiment (left). The response of IPCs to an addition of 20 mM leucine is abolished when animals were fed prior to the experiment (right). (D) Leucine-induced neuronal activity of IPCs is abolished after *Mnd* knockdown (*Dilp2*-Gal4 > UAS-*GCaMP3*;UAS-*Mnd*^dsRNA^). (E) Representative images showing calcium activation by 20 mM leucine in IPCs of the control genotype (*Dilp2*-Gal4 > UAS-*GCaMP3*) and after the *Mnd* knockdown. The statistics in (C) and (D): *^∗^*p *<* 0.05 and ^∗∗^p *<* 0.01: significant difference from control (t test or Mann-Whitney test); the data are mean ± SEM. The scale bar represents 30 μm.

Leucine, isoleucine, and valine are three branched-chain amino acids (BCAAs; Figure 1A). Out of those BCAAs, leucine and isoleucine directly stimulate the increase of free cytosolic Ca^2+^ in mammalian β-cells, which in turn leads to insulin secretion into the blood to regulate sugar metabolism (Bolea et al., 1997, Newsholme et al., 2005). Cheng et al. (2016) recently proposed that LAT1 could be critical for this insulin release. In *Drosophila*, leucine is also involved in the release of DILP2 from IPCs. But up to now only an indirect effect of leucine had been suggested; it was thought that detection by IPCs requires an intermediate hormonal signal (Géminard et al., 2009).

To shed new light on IPC amino acid sensing, we first wondered whether BCAAs could also directly stimulate the neuronal activity of IPCs in *Drosophila.* To answer this, an ex vivo brain preparation was set up in which the neuronal activity of IPCs can be monitored when BCAAs are applied. GCaMP3 was expressed using *Dilp2*-Gal4 and UAS-*GCaMP3* transgenes, which report neuronal activity upon binding of intracellular Ca^2+^ (Tian et al., 2009). We investigated the third-instar larval stage, since *Drosophila* feed the most during this specific stage of development to increase their body size 3-fold in just 2 days at 25°C (Ghosh et al., 2013). Larval fillet preparations were made to directly access the brain. The digestive tract and the fat body were removed to avoid any hormonal communication between these peripheral tissues and brain IPCs. The brain was then bathed in a hemolymph-like solution (HL6), with or without BCAAs, and neuronal activity of IPCs was simultaneously monitored using a fluorescent microscope (Figure 1B).

Under these conditions, brains from starved larvae (*Dilp2-*Gal4 > UAS-*GCaMP3*) displayed a robust increase in IPC neuronal activity when 20 mM leucine or 20 mM isoleucine was added to the HL6 solution. In contrast, no increase was observed when either 20 mM valine or 2 mM glutamate or HL6 solution without amino acids was applied. When animals were fed with a rich diet instead of starved, the enhancement in IPC neuronal activity did not occur (Figures 1C and 1E).

These data demonstrate that, similar to their mammalian counterpart the pancreatic β-cells, *Drosophila* brain IPCs are capable of directly sensing BCAAs such as leucine and isoleucine without the need of any hormonal signal coming from peripheral tissues.

### Expression of the System-L Transporter MND in IPCs

In mammals, LAT1 (SLC7A5) and LAT2 (SLC7A8) are two known system-L transporters responsible for the transport of large neutral amino acids including leucine (Kanai et al., 1998, Pineda et al., 1999). The *Drosophila* genome encodes two LAT1-like transporters, including MND (Reynolds et al., 2009).

**Figure 2:**
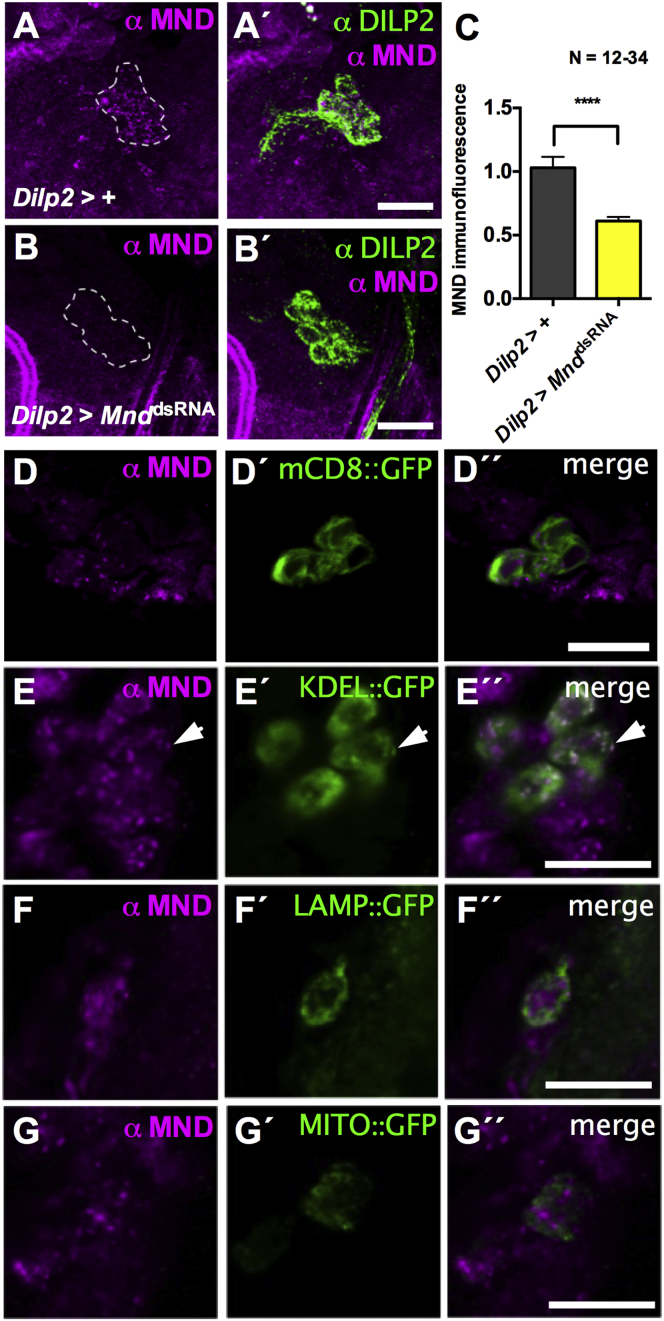
MND Leucine Transporter Is Expressed in Larval IPCs (A) Representative confocal images of whole mount larval IPCs labeled by anti-DILP2 (green) and anti-MND (magenta). (B) *Mnd* RNAi mediated by *Dilp2*-Gal4 reduces MND antibody signal. (C) Quantification of anti-MND immunoreactivity in IPCs. (D–G) Representative confocal images of larval IPCs expressing various intracellular compartment markers fused to GFP. (D) Anti-MND immunoreactivity partially co-localizes with the plasma membrane mCD8::GFP expression driven by *Dilp2*-Gal4. (E) Anti-MND signal overlaps with the ER marker (KDEL::GFP) driven by *Dilp2*-Gal4 (arrow). (F and G) Anti-MND signal does not overlap with a lysosomal marker (LAMP::GFP) (F) or a mitochondrial marker (MITO::GFP) driven by *Dilp2*-Gal4 (G). *^∗∗∗∗^*p *<* 0.0001: significant difference from control (t test); the data are mean ± SEM. The scale bar represents 20 μm.

We wondered if MND is expressed in larval brain IPCs. A rabbit polyclonal anti-MND antibody was generated. In whole mount brains, robust localization of MND in IPCs could be observed in control larvae (Figure 2A). This signal was drastically diminished after RNAi-mediated knock down of *Mnd* specifically in IPCs (*Dilp2*-Gal4 > UAS*-Mnd*^dsRNA^) confirming the antibody specificity (Figures 2B and 2C).

As shown by the arrowhead in Figure 2E, anti-MND labeling in IPCs overlaps with an ER marker (KDEL::GFP). This co-localization mostly corresponds to punctate staining within IPCs that is strongly diminished after knock down of *Mnd* (Figures 2B and 2E). We could not detect strong co-staining using a plasma membrane tethered GFP (mCD8::GFP), a lysosomal marker (LAMP::GFP), or a mitochondrial marker (MITO::GFP) (Figures 2D, 2F, and 2G). When using a fat body Gal4 driver (*OK376*-Gal4), co-localization between MND and GFP was consistently detected with the ER marker (KDEL::GFP), but also clearly with the plasma membrane tethered GFP (mCD8::GFP; Figure S1). This suggests that MND might be able to be sent to the plasma membrane from the ER.

These results indicate that MND is expressed in larval brain IPCs, and that it seems to localize predominantly to the ER. We cannot exclude that low amounts of MND could go to the plasma membrane in IPCs, since in other tissues (e.g., the fat body) such localization is possible.

### MND Mediates the Leucine Control of IPC Neuronal Activity

Since MND is a confirmed leucine transporter (Reynolds et al., 2009), we wondered whether it was required for extracellular leucine to stimulate IPC activity.

To reveal a putative function of *Mnd* in IPCs, we could not use a mutant because *Mnd* is expressed in several tissues and leads to general developmental problems in mutant larvae (Martin et al., 2000; http://flybase.org/reports/FBgn0002778.html). To solve this issue, *Mnd*-specific knockdown in IPCs was induced using a *Dilp2*-Gal4 driver. In parallel, GCaMP3 was co-expressed to measure neuronal activity. Larval IPCs in which *Mnd* has been downregulated by RNAi (*Dilp2-*Gal4 > UAS*-GCaMP3*;UAS*-Mnd*^dsRNA^) no longer show enhancement in neuronal activity when 20 mM leucine was applied (Figures 1D and 1E).

This result indicates that MND is necessary for the direct effect of leucine on IPC activity.

### MND Affects DILP Secretion in Cultured Brains

We next wanted to test whether MND acts not only on IPC activity, but also on DILP secretion. From the work of Géminard et al. (2009), we assume that DILP disappearance in IPCs corresponds to its release in the extracellular medium.

**Figure 3:**
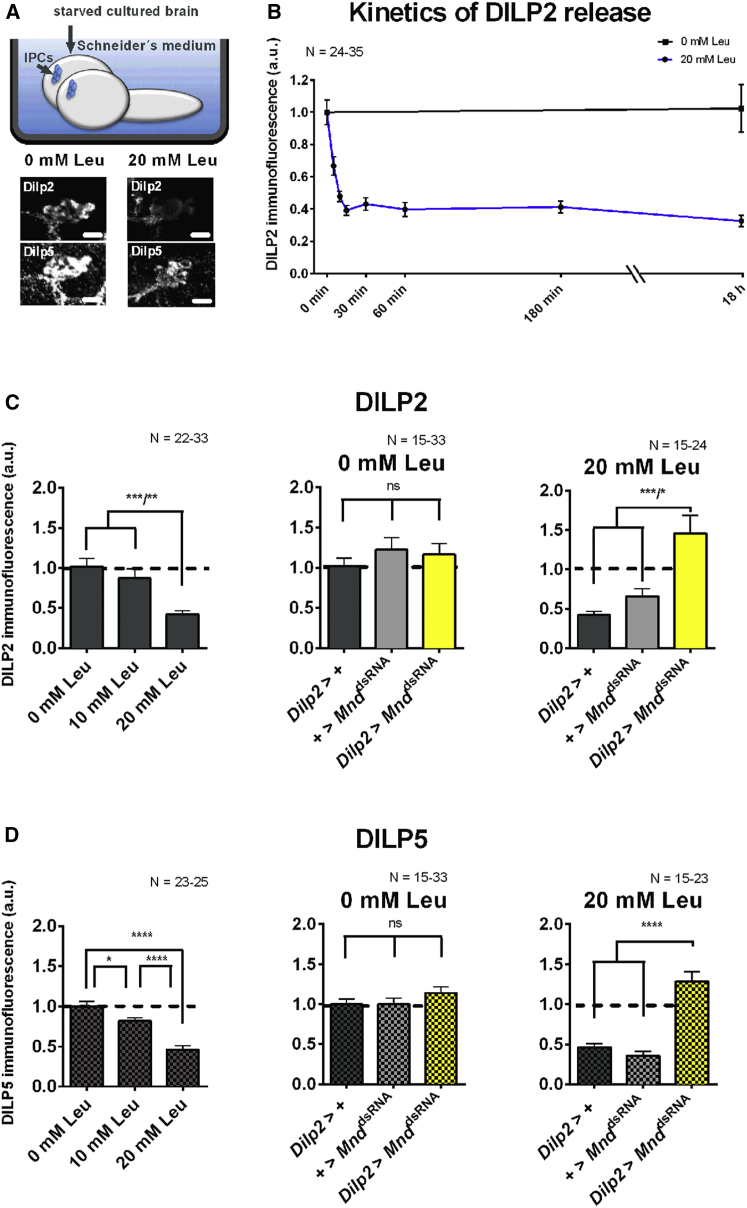
Brains Sense Leucine Autonomously (A) Top, isolated brains of starved larvae were incubated in Schneider’s *Drosophila* medium supplemented or not with leucine. The representative confocal stack images of IPCs visualized by anti-DILP2 or anti-DILP5 are shown (bottom). The scale bar represents 20 μm. (B) Kinetics of DILP2 release in the control genotype (*Dilp2*-Gal4 > +) upon incubation of cultured brains in Schneider’s medium without additional leucine (0 mM leucine) or with a supplementation of 20 mM leucine. (C) Left, quantified immunofluorescence intensities for DILP2 upon increasing levels of additional leucine in Schneider’s medium in the control genotype (*Dilp2*-Gal4 > +). The quantified DILP2 immunofluorescence intensities of isolated brains incubated in Schneider’s medium supplemented or not with 20 mM leucine are shown (middle and right). The control genotypes (*Dilp2*-Gal4 or UAS-*Mnd*^*dsRNA*^) are represented by gray bars. The larvae in which the expression of *Mnd* is downregulated in IPCs are represented by yellow bars. (D) DILP5 immunofluorescence using the same conditions and genotypes as in (C). Not significant, ns; ^∗^p *<* 0.05; *^∗∗∗^*p *<* 0.001; and *^∗∗∗∗^*p *<* 0.0001: significant difference between genetic controls and *Mnd* knockdown animals (one-way ANOVA followed by a Bonferroni post hoc test or Kruskal-Wallis test followed by a Dunn’s post hoc test); the data are mean ± SEM.

Brains from starved larvae of our control genotype (*Dilp2-*Gal4 > +) were dissected and cultured in Schneider’s medium supplemented with various amounts of leucine for 18 hr. The amount of stored DILP2 in IPCs was revealed by anti-DILP2 staining. As expected, intracellular DILP2 levels were high in brains, which were incubated in Schneider’s medium without additional leucine. On the other hand, brain incubation in Schneider’s medium supplemented with 20 mM leucine robustly induced the secretion of DILP2 from IPCs, which is indicated by low DILP2 signal intensities (Figures 3A–3C).

The kinetics of DILP2 release were next determined using Schneider’s medium supplied with 20 mM leucine. IPCs from brains incubated 5 min, 10 min, or 15 min using this medium showed a continuous drop of DILP2 level. The release of Dilp2 by 20 mM leucine was already apparent after 5 min of incubation time and reached a maximum effect after 15 min. Longer incubation time did not further reduce DILP2 signal (Figure 3B, blue line). This effect on DILP2 secretion from IPCs is dependent on leucine, since the incubation with a regular Schneider’s medium has no effect on DILP2 secretion over 18 hr (Figure 3C, black line). The drop of DILP2 levels observed in Figures 3B and 3C are therefore due to the presence of a high extracellular concentration of leucine.

We next tested whether this DILP2 release from IPCs in cultured brains was affected when *Mnd* was specifically downregulated in these cells. Ex vivo cultured brains of our two controls (*Dilp2-*Gal4 > + or + > UAS*-Mnd*^dsRNA^) and *Mnd* knockdown (*Dilp2-*Gal4 > UAS*-Mnd*^dsRNA^) show robust staining of DILP2 within IPCs when incubated in regular Schneider’s medium during 18 hr. Adding 20 mM leucine to the culture medium led to a strong decrease in DILP2 levels within IPCs of both controls, but not after *Mnd* knockdown (Figure 3C). We observed a very similar leucine-dependent loss of DILP5 from IPCs (Figure 3D).

These data show that MND is necessary for leucine-dependent loss of DILP2 and DILP5 from IPCs. We assume that this loss represents DILP secretion.

### The GDH-Pathway Mediates the Leucine Effect on DILP Secretion from IPCs

**Figure 4:**
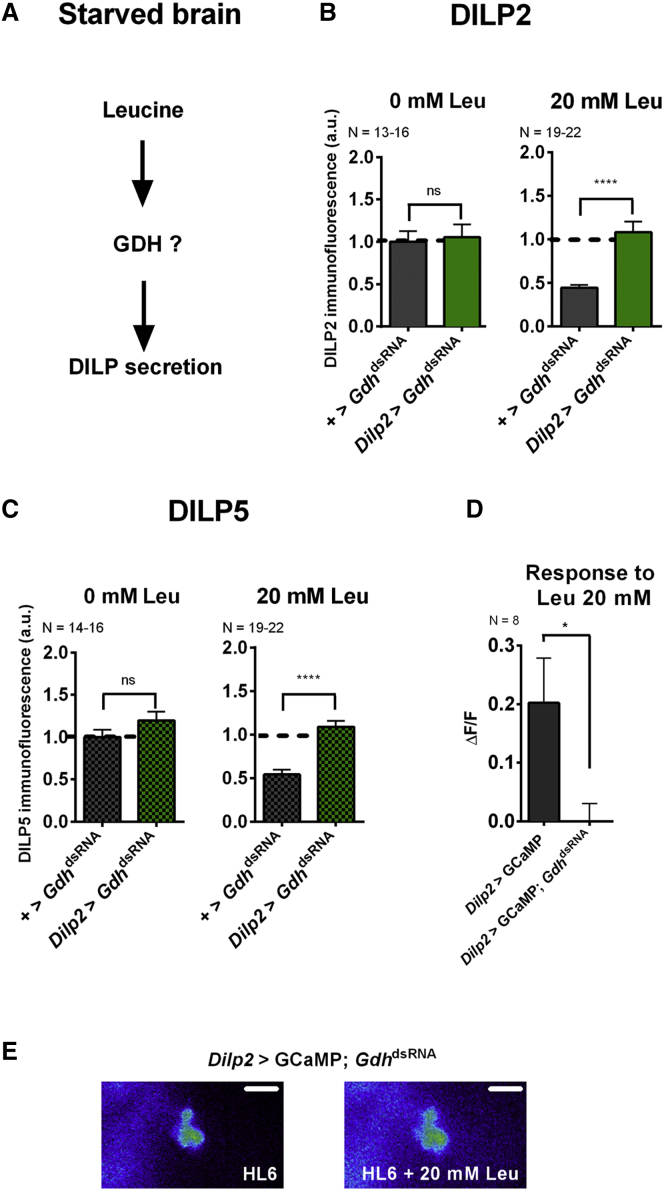
Leucine Sensing in the Brain IPCs Requires the GDH-Pathway (A) Which pathway could mediate the leucine control of DILP release from IPCs? (B) Quantified DILP2 immunofluorescence intensities of isolated brains of control larvae (control in gray; knockdown genotype, green). (C) Same conditions as in (B), but showing DILP5 immunofluorescence. (D) Leucine-induced activation of the IPCs by a hemolymph-like solution in which leucine was directly added to starved larvae was revealed by Ca^2+^-imaging via GCaMP3. (E) Representative images of GCaMP3 and *Gdh* RNAi in IPCs show no enhanced neuronal activation after the application of 20 mM leucine. The scale bar represents 30 μm. Statistics: not significant, ns; *^∗^*p *<* 0.05; and ^∗∗∗∗^p < 0.0001: significant difference between genetic control and knockdown genotype (t test or Mann-Whitney test); the data are mean ± SEM.

Leucine is known to activate two different pathways in mammals. One involves TOR, the other GDH (Cheng et al., 2016, Lynch, 2001, Lynch et al., 2000, Zhou and Thompson, 1996). Both have already been described as connections between nutrient availability, metabolism, and growth and might also be involved in linking the effect of leucine on DILP release in brain IPCs of *Drosophila* larvae (Figure 4A) (Cook and Morley, 2007, Géminard et al., 2009, Kim et al., 2008).

In mammalian cells, leucine promotes the assembly of the nutrient responsive TOR complex 1 (TORC1) and thereby positively affects its activation (Durán et al., 2012, Sancak et al., 2008, Sancak et al., 2010). The TORC1 inhibitor rapamycin reduces insulin secretion from pancreatic β-cells (Fraenkel et al., 2008). To test whether TORC1 is involved in leucine-dependent DILP2 release from *Drosophila* IPCs, the expression of *Raptor* was downregulated. RAPTOR is an essential member of TORC1 and is absolutely required for leucine sensing (Kim et al., 2002). Brains from starved control larvae (*Dilp2*-Gal4 > +) or knockdown larvae in which we specifically drove a *Raptor*^dsRNA^ in IPCs were cultured. This targeted inactivation of TORC1 had no effect on the leucine-dependent release of DILP2 from IPCs. This result was confirmed by overexpressing a dominant-negative form of TOR (TOR^TED^) in IPCs. Cultured brains from this genotype did not show any impairment of leucine-induced DILP2 secretion (Figure S2A). DILP5 secretion under the same conditions and with the same tools could not be increased (Figure S2B). Thus, we could not show that the TOR pathway is involved in DILP release by brain IPCs.

DILP2 and DILP5 secretion could be mediated by the GDH pathway. GDH catalyzes the transformation of glutamate to alpha-ketoglutarate (α-KG) and can allosterically be activated by leucine (Hudson and Daniel, 1993). α-KG is used in the Krebs cycle and is ultimately important for ATP generation. In β-cells, a rise in the ATP/ADP ratio contributes to the depolarization of the plasma membrane, which leads to insulin secretion (Gao et al., 2003). To test if the GDH pathway can link leucine to DILP2 secretion from *Drosophila* IPCs, the expression of GDH was specifically downregulated in these cells (*Dilp2*-Gal4 > UAS*-Gdh*^dsRNA^). As expected, cultured brains of control genotype larvae secreted DILP2 when Schneider’s medium was supplied with leucine. In contrast, IPCs of *Gdh* knockdown larvae were unresponsive to the application of leucine and intracellular DILP2 levels remained high compared to controls (Figure 4B). Similar effects of *Gdh* knockdown were observed for DILP5 secretion (Figure 4C).

These results were confirmed by imaging IPC activity. While IPCs of control animals (*Dilp2*-Gal4 > UAS*-*GCaMP3 in Figures 1D and 1E) showed a robust increase in their activity when bathed with 20 mM leucine, GCaMP3 fluorescence remained at background level after *Gdh* knockdown (Figures 4D and 4E).

These data show that *Gdh* expression is necessary for the leucine-dependent increase in IPC activity leading to DILP2 and DILP5 release.

### MND Controls In Vivo Leucine-Dependent DILP Release from IPCs

**Figure 5:**
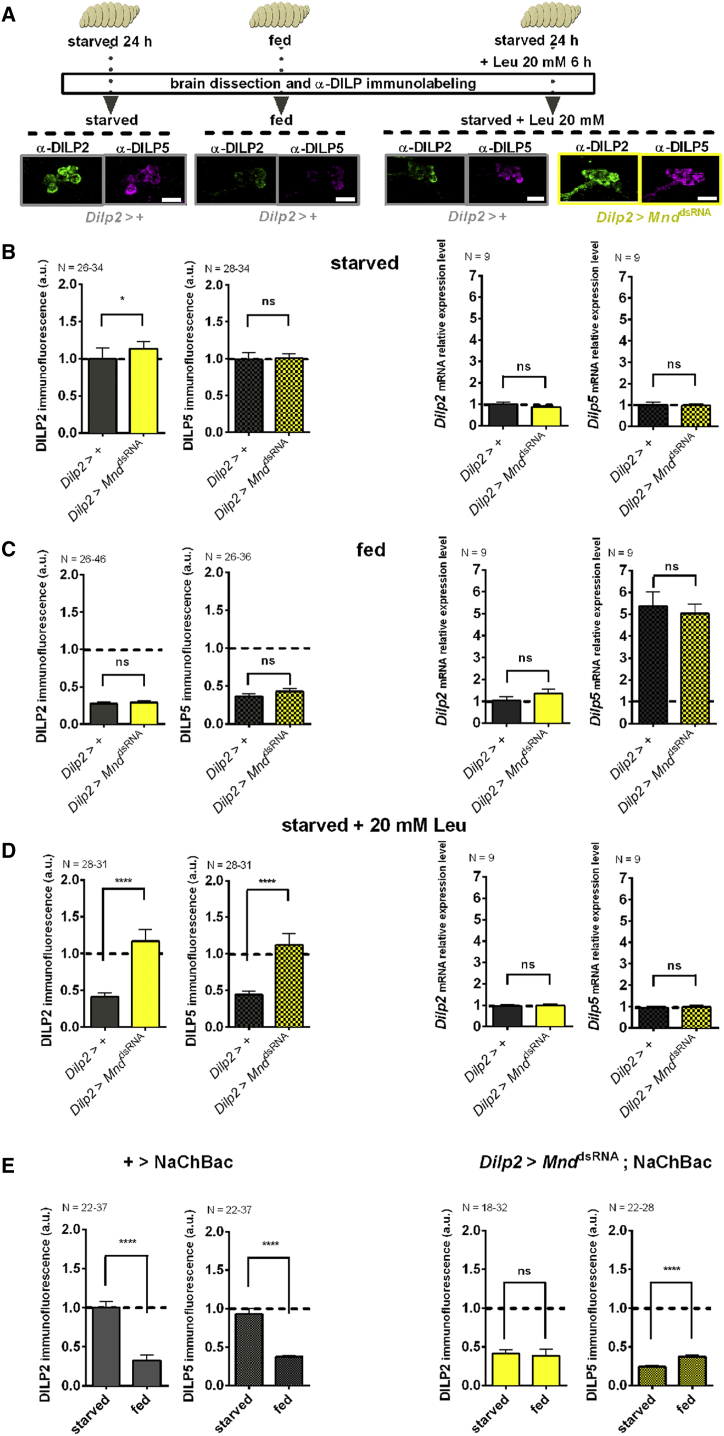
MND Controls Leucine-Induced Release of DILP from IPCs in Starved Larvae (A) Top, third-instar larvae in feeding stage were either starved for 24 hr on PBS + 1% agar and 1% sucrose (starvation medium), fed with regular diet, or starved for 24 hr and fed with only 20 mM leucine added to the starvation medium prior to brain dissection. The intracellular DILP2 and DILP5 levels in IPCs were visualized by anti-DILP2 (green) and anti-DILP5 (magenta). (B) Left, DILP2 (filled bars) and DILP5 (squared bars) immunofluorescence intensities for *Dilp2*-Gal4 > + control larvae (gray bars) and larvae expressing a *Mnd*^*dsRNA*^ in IPCs (yellow bars, *Dilp2*-Gal4 > UAS-*Mnd*^*dsRNA*^). mRNA levels of *Dilp2* and *Dilp5* are quantified under the same conditions and genotypes (right). (C) Identical to (B) except that animals are fed with a regular diet. (D) Identical to (B) except that animals are starved and then fed with a minimal diet supplied with 20 mM leucine. (E) Left, immunofluorescence intensities for DILP2 and DILP5 show that the release of these DILPs is mediated by MND and are dependent on the feeding status of the larva in a control genotype (+ > UAS-*NaChBac*). The simultaneous expression of NaChBac and *Mnd*^*dsRNA*^ by *Dilp2*-Gal4 leads to constant activation and low intracellular levels of both DILP2 and DILP5 immunofluorescence in starved animals (*Dilp2*-Gal4 > UAS-*Mnd*^*dsRNA*^;UAS-*NaChBac*) (right). Statistics: not significant, ns; *^∗^*p *<* 0.05; and *^∗∗∗∗^*p *<* 0.0001 (t test, or Mann-Whitney test). All data are mean ± SEM.

We next wanted to determine whether MND is involved in the sensing of leucine from IPCs in vivo, and what the impact of MND-dependent DILP2 and DILP5 release is in a physiological context. IPC DILP2 and DILP5 levels in intact wild-type larvae vary depending on the feeding status. This variation is not due to a change in the expression level of these DILPs. Rather, the variation is due to enhanced DILP release upon feeding (Géminard et al., 2009). We used the anti-DILP2 and anti-DILP5 antibodies to compare DILP2 and DILP5 levels in IPCs of intact larvae that were in various feeding states (Figure 5A). A low nutrient food medium containing only 1% sucrose, PBS, and agar served as a “starvation medium”. Keeping animals on this minimal food source for 24 hr led to an expected accumulation of DILP2 and DILP5 in IPCs in both the control genotype (*Dilp2*-Gal4 > +) and *Mnd* knockdown genotype (starved condition; Figure 5B). A reduction of this DILP2 and DILP5 immunolabeling could be observed when larvae were fed on a rich food medium containing amino acids, fatty acids, and sugars in both genotypes (fed condition; Figure 5C). To test the specific effect of leucine, larvae were starved for 24 hr and then fed for 6 hr with starvation medium supplemented with 20 mM leucine. Control larvae showed a significant reduction in IPC intracellular DILP2 and DILP5 levels when fed on this diet. Strikingly, this release of both DILPs from IPCs was totally lacking in *Mnd* knockdown larvae (starved + Leu 20 mM condition; Figure 5D). Since the expression level of *Dilp2* mRNA did not vary among feeding conditions or genotypes (Figures 5B–5D, right histograms), we conclude that MND is a key actor for leucine-dependent release of both DILP2 and DILP5 from IPCs in vivo. This provides additional evidence that leucine coming from the diet triggers DILP secretion from IPCs in an MND-dependent manner.

We also verified that downregulation of *Mnd* expression specifically affects the ability to sense leucine, and that IPCs can release both DILP2 and DILP5 when they are forced to do so. For this purpose, the bacterial Na^+^ channel (NaChBac) was co-expressed together with the *Mnd*^dsRNA^ construct specifically in IPCs. NaChBac leads to a constant activation of neurons by importing sodium and should therefore cause constant release of DILPs (Luan et al., 2006). In contrast to the control genotype (+ > UAS-*NaChBac*), DILP2 and DILP5 release from *Mnd* knockdown larvae expressing NaChBac in IPCs (*Dilp2*-Gal4 > UAS-*Mnd*^dsRNA^;UAS-*NaChBac*) was no longer dependent on the feeding status. This indicates that MND specifically controls leucine sensing, but does not affect general IPC functions such as the ability to release DILPs (Figure 5D).

Taken together, these data provide in vivo evidence that MND is required for detection of dietary leucine, which then triggers DILP secretion from IPCs.

Despite our intense efforts, including western blot, dot blot, and enzyme immunoassays, we could not measure any modification in DILP2 or DILP5 hemolymph levels under the feeding conditions we tested (Figures S3 and S4). This suggests that either the antibodies are not specific enough to detect such variations, that circulating DILPs are masked by binding proteins, or that the diet-induced changes in hemolymph levels of these two DILPs are too low to be detected. We favor the latter hypothesis, given that DILPs in IPCs are highly concentrated, but would not be once released into the hemolymph.

### Leucine Signaling through MND in Larval IPCs Regulates Downstream Metabolic Pathways

**Figure 6:**
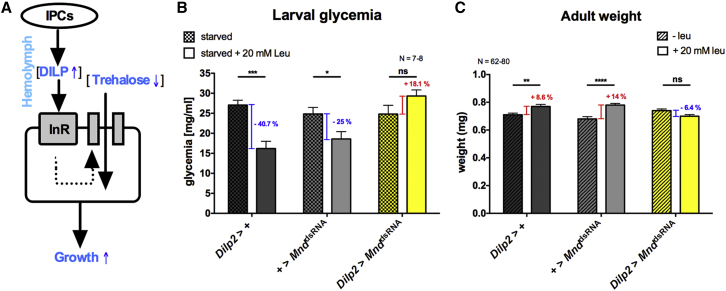
MND Related Leucine Signaling in Larval IPCs Regulates Downstream Metabolic Pathways (A) The IPCs release DILPs into the hemolymph upon feeding. They bind to their receptor (InR). They then induce the uptake of sugars like trehalose and glucose from the hemolymph, plus promote growth. (B) Hemolymph trehalose and glucose levels were determined under different starvation conditions in control larvae (gray bars) and when MND expression was downregulated (yellow bars). (C) The weight of newly hatched adult males was measured in the same genotypes as in (B) when animals were raised either on a poor medium supplied with 20 mM leucine or on only a poor medium. Statistics: not significant, ns; *^∗^*p < 0.05; ^∗∗^p < 0.01; ^∗∗∗^p < 0.001; and ^∗∗∗∗^p < 0.0001: significant difference between feeding conditions (two-way ANOVA followed by a Bonferroni post hoc test); the data are mean ± SEM.

In mammals, insulin secretion into the blood leads to increased uptake of circulating glucose into muscle and adipocytes through the insulin responsive glucose transporter 4 (GLUT4) (Czech, 1995, Kono, 1983). This glucose is then stored or used as a source of energy. In *Drosophila*, previous studies have shown that DILPs regulate the level of the most abundant sugar in the hemolymph, which is trehalose (Figure 6A) (Grönke et al., 2010, Wyatt and Kale, 1957). Since leucine regulates DILP2 and DILP5 release, feeding larvae with a diet containing leucine should also impact the level of trehalose in the hemolymph and should have a consequence on larval growth.

To test this hypothesis, we measured the hemolymph sugar level from groups of larvae that were either starved or starved and then fed with a minimal medium supplied with 20 mM of leucine. In the two control genotypes (*Dilp2-*Gal4 > +, and + > UAS*-Mnd*^*dsRNA*^), sugar levels were reduced after feeding the larvae with a poor medium (see Experimental Procedures for the composition, “Weight Determination”) supplied with 20 mM leucine. In contrast, the hemolymph sugar level of *Mnd* knockdown larvae (*Dilp2*-Gal4 > UAS-*Mnd*^dsRNA^) remained stable under the same conditions (Figure 6B).

Next, we tested the effects of MND-dependent DILP2 and DILP5 release on larval growth. As expected, control larvae fed a minimal medium with 20 mM leucine grew to become significantly bigger adults compared to larvae fed the same medium without leucine. In contrast, larvae produced adults of similar weight when *Mnd* expression in IPCs was downregulated by RNAi, whether leucine was included in the medium or not (Figure 6C).

These results show that MND is required for leucine to regulate levels of hemolymph sugars and growth in *Drosophila*.

## Discussion

### Insulin Release Relies on Direct Leucine Sensing

Previous work studying the relationship between feeding and DILP signaling in *Drosophila* proposed communication via multiple hormonal signals between peripheral tissues such as the fat body or the gut and a specific subset of DILP producing neurons (IPCs) located within the larval brain. This model proposes that the *Drosophila* fat body and/or the gut are sensing the availability of nutrients such as amino acids, fatty acids, or sugars. They in turn secrete hormonal factors into the hemolymph, which stimulate or inhibit the activity of IPCs, thus controlling DILP secretion (Géminard et al., 2009, Koyama and Mirth, 2016, Rajan and Perrimon, 2012, Ren et al., 2015, Sano et al., 2015).

In mammals, it has been long recognized that IPCs, pancreatic β-cells, are also directly sensing nutrients such as amino acids (Bolea et al., 1997, Newsholme et al., 2005). Especially, the essential branched-chain amino acids leucine and isoleucine were found to acutely stimulate insulin secretion (Sener et al., 1981). However, up to now there was no evidence of a direct action of leucine on insulin-secreting cells in *Drosophila*.

Here, we report that *Drosophila* IPCs increase their neuronal activity after exposure to extracellular branched-chain amino acids, even if the peripheral tissues such as the fat body and the gut have been removed. Similar to the situation in mammalian β-cells, leucine leads to higher activity than isoleucine in *Drosophila* IPCs. The third branched-chain amino acid, valine, does not significantly affect cell physiology either in mammals or in *Drosophila* (Figure 1C) (Sener et al., 1981).

Acting in parallel to the indirect hormonal signals, this direct pathway represents a faster mechanism to regulate IPC activity. Our data on isolated brain cultures show that a remarkable amount of the DILP2 store is already secreted 5 min after the brains were immersed in leucine-enriched medium. The intracellular DILP2 level reaches a minimum after 15 min of incubation and does not recover as long as the brains stay in a leucine rich environment. Therefore, direct leucine sensing may serve as an effective way to signal the availability of amino acids after food deprivation.

Géminard et al. (2009) also followed a DILP2 release curve using whole larvae. They showed that DILP2 in starved animals is released much slower and is still decreasing 2 hr after refeeding with a regular diet. This slower release compared to our results probably reflects the additional time required for dietary nutrients, including leucine, to be taken up by the gut, metabolized, and released into the hemolymph before entering into the brain where they could directly act on IPCs.

Our results suggest that direct sensing of nutrients such as leucine by IPCs is a conserved mechanism. It exists in parallel to hormonal cross talk between peripheral organs and IPCs in mammals and in *Drosophila.* Such a direct pathway might provide a faster response to the intake and use of nutrients after starvation.

It is also possible that leucine detection might occur via sNPF neurons. sNPF released from neurons adjacent to the IPC regulate DILP secretion and growth through sNPF receptors on the IPC, via ERK signaling (Lee et al., 2008). Thus, leucine might be detected by the sNPF-producing neurons or associated glial cells, and this could indirectly modulate DILP secretion from IPCs in an MND-dependent manner. While this seems unlikely since MND is an amino acid transporter, this alternative pathway or the possibility that it exists in parallel with direct detection of leucine by MND cannot be ruled out.

### GDH Activity Is Required for DILP Release In *Drosophila*

**Figure 7:**
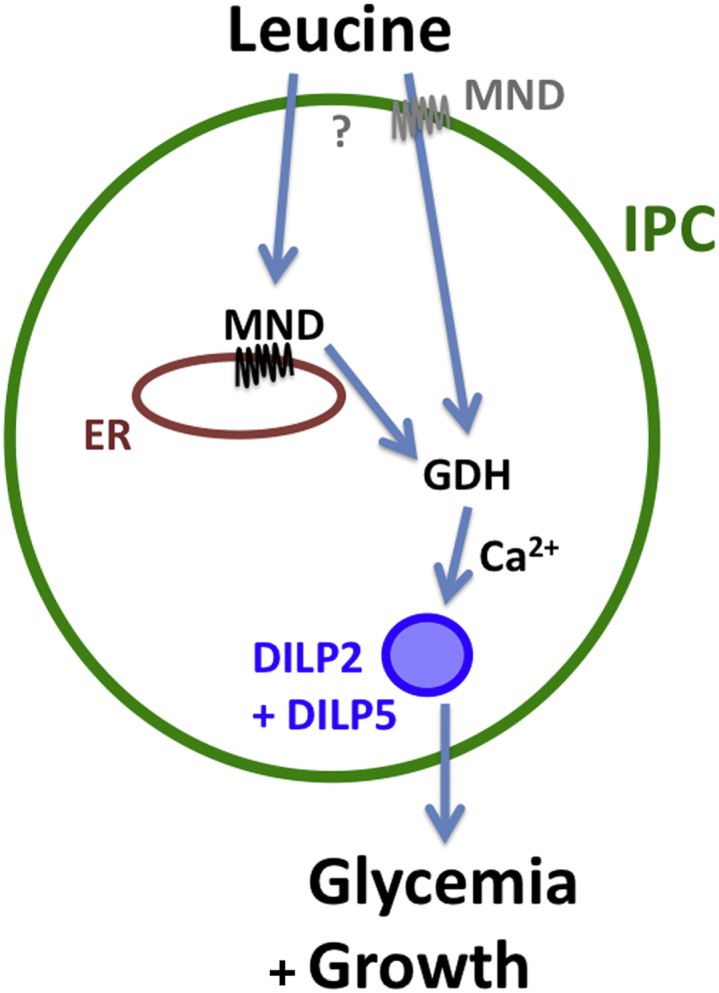
A Model for the Direct Sensing of Leucine through MND and GDH in IPCs Leucine acts on the IPCs activity via two possible pathways using MND and the GDH pathway and consequently controls DILP2 and DILP5 release into the hemolymph to affect glycemia and growth.

Our data show that leucine needs the LAT1 homolog MND to act on IPCs. Very recently, Cheng et al. (2016) proposed that LAT1 is required for regulating cell signaling and function in β-cells. Therefore, MND appears to represent a conserved element between *Drosophila* and mammals for leucine sensing on IPCs. In mammals, GDH is known to play a role in insulin release, and intracellular leucine is an allosteric activator of GDH. Once activated, GDH converts glutamate into alpha-ketoglutarate, which enters the Krebs cycle and ultimately leads to increased production of ATP during aerobic phosphorylation. Increasing intracellular ATP concentrations in mammalian pancreatic β-cell leads to the closure of an ATP-sensitive potassium channel followed by the depolarization of the cell membrane and consequently to insulin release (Göhring and Mulder, 2012, Petit and Loubatières-Mariani, 1992, Sener and Malaisse, 1980, Sener et al., 1981). In *Drosophila*, it is possible that increased ATP also leads to the enhanced activity of brain IPCs, finally leading to the DILP2 and DILP5 release that we observed (Figure 7). In contrast, we could not identify a clear involvement of the TOR pathway on DILP2 and DILP5 release from IPCs in *Drosophila*. Thus the activation of TOR in β-cells might represent a specific feature in mammals.

## Experimental Procedures

### *Drosophila* Strains and Food

Fly strains used in this study were *w*^*1118*^ and *Dilp2*-Gal4 (Brogiolo et al., 2001, Rulifson et al., 2002); UAS-*mCD8GFP*, UAS-*Tor*^*TED*^, UAS-*NaChBac*, UAS-*MitoGFP*, UAS-*LampGFP*, and UAS-*KdelGFP* (Bloomington Stock Center); UAS-*Gdh*^*dsRNA*^, UAS-*Mnd*^*dsRNA*^, and UAS-*Raptor*^*dsRNA*^ (VRDC); and UAS-*GCaMP3.0* (gift from Richard Benton, UNIL).

All strains have been backcrossed to an isogenic *w*^*1118*^ strain for five generations. Animals were reared on *Drosophila* standard corn/yeast medium at 25°C. Larvae were fasted on starvation medium containing 1% agar and 1% sucrose in PBS (Géminard et al., 2009). The media were boiled to solubilize the agar. Leucine supplemented media were cooled down to 65°C before its addition.

### Calcium Imaging

The composition of HL6 (Macleod et al., 2002) was modified to replace BCAAs by glutamine: 23.7 mM NaCl, 24.8 mM KCl, 24.8 mM MgCl_2_, 10 mM NaHCO_3_, 20 mM isothionic acid Na^+^, 5 mM BES, 80 mM trehalose, 5.7 mM L-alanine, 2 mM L-arginine-HCl, 14.5 mM glycine, 12.3 mM L-glutamine, 11 mM L-histidine, 1.7 mM L-methionine, 13 mM L-proline, 2.3 mM L-serine, 2.5 mM L-threonine, 1.4 mM L-tyrosine, 0.0001 mM TPEN, and 1 mM Trolox (all from Sigma-Aldrich), pH 7.2.

L1 larvae were collected 24 hr after egg laying (4 hr egg collections) and reared at a density of 30 larvae/tube at 25°C until they have reached the feeding third-instar larval state. Larvae were starved for 24 hr in starvation medium and washed in PBS prior to the experiment. To expose the brain, “filet preparations” were obtained as previously described (Brent et al., 2009). The dissection was done in HL6 lacking Ca^2+^. Peripheral tissues such as the fat body and the digestive tract were removed. To avoid any micromovement of the brain during the experiment dissection, small pins were placed on each side of the brain. Before imaging, HL6 medium without Ca^2+^ was replaced by 0.25 mL HL6 medium with 0.5 mM Ca^2+^. During the experiment, 0.25 mL of either HL6 medium + 0.5 mM Ca^2+^ (control) or HL6 medium + 0.5 mM Ca^2+^ supplemented with 2× BCAA (L-Leucine or L-Isoleucine or L-Valine; Sigma-Aldrich) were added to have a final concentration of 1× BCAA. GCaMP3 fluorescence was viewed with Leica DM6000B microscope under a 25× water objective. GCaMP3 was excited using a Lumencor light engine supplied with diodes of 485 ± 25 nm. Emitted light was collected through a 505–530 nm band-pass filter. Leica MM AF 2.2.0 was used for data collection and acquisition. Images were acquired at 250 ms per frame at resolution of 256 × 256 using an Orca-Flash 4.0 camera. For each experiment, 480 images were taken; 120 before the application of additional BCAAs (30 s) and 360 after (90 s). The ten first frames before the BCAA application were used to establish the base line F. Adjacent regions to the region of interest were used to determine the autofluorescent background level. Changes in fluorescence versus the initial fluorescence (%ΔF/F) were calculated as (the peak fluorescence after t = 120 frames minus F versus F) × 100 (Miyamoto et al., 2012).

### Brain Cultures

Brain cultures were performed as previously described, with minor modifications (Britton and Edgar, 1998, Géminard et al., 2009). Briefly, larvae were reared on standard medium and starved on starvation medium 24 hr. Larvae were then sterilized by a 30 s washing step in ethanol (70%) and rinsed with sterile water. Brains were then dissected in Schneider’s *Drosophila* medium (Pan, Biotech) in sterile conditions using sterilized tools. Brains were transferred into a 4-well plate containing either 1 mL of Schneider’s *Drosophila* medium (control) or 1 mL of Schneider’s *Drosophila* medium supplemented with leucine. Cultured brains were incubated at 25°C for 18 hr.

### Immunohistology

Primary antibodies used were rat anti-DILP2, rabbit anti-DILP5 (1/800; Géminard et al., 2009), and mouse anti-GFP (1:100, G6539, Sigma-Aldrich). Polyclonal anti-MND antibody was produced by immunization of rabbits with a synthetic peptide (MRYKQPKTERPIKVN) corresponding to the last cytoplasmic loop of MND. This anti-MND antibody was used at 1:250. Secondary antibodies (anti-rat IgG-Alexa Fluor 594, anti-mouse IgG-Alexa Fluor 488, and anti-rabbit IgG-Alexa Fluor 594) were obtained from Thermo Fisher Scientific and used at 1:400.

### Whole-Mounted Larval Brains

Larval brains were dissected in PBS, fixed in 4% paraformaldehyde for 45 min at room temperature (RT), and washed for 6 × 10 min in PBS + 0.3% Triton X-100 (PBS-T) and 1 × 10 min in PBS + 1% Triton X-100. Tissues were blocked in PBS-T containing 10% normal goat serum (NGS; Sigma #G9023) for 1 hr at RT. Primary antibodies were diluted in PBS-T + 5% NGS and allowed to incubate with the tissues over night at 4°C. After washing 6 × 10 min in PBS-T, samples were labeled with the appropriate secondary antibody at 1:400 in PBS-T containing 5% NGS for 3 hr at RT. They were washed for 6 × 10 min in PBS and mounted in Vectashield mounting medium (Vector Laboratories). Fluorescence was observed using a confocal microscope (Leica TCS SP2 or Zeiss LSM 780).

### Cross-Sections of Larval Brains

For sectioning, larvae were cut in half (transverse section) and fixed in 4% paraformaldehyde (PFA) in PBS pH 7.4 for 3 hr at 4°C. The fixative was then replaced by 25% sucrose in *Drosophila* Ringer’s solution and incubated overnight at 4°C. Larval brains were dissected and embedded in Tissue-Tek (Sakura Finetek), frozen in liquid nitrogen, and sectioned at 14 μm. Section were washed 2 × 10 min with TBS + 0.01% Triton (TBS-T) and blocked with 1% normal goat serum for 30 min at RT. Primary antibodies were diluted in blocking solution and incubated with the samples overnight at 4°C. Sections were washed 2 × 10 min with TBS-T and incubated with the appropriate secondary antibodies diluted in blocking solution and incubated with the samples for 3 hr at RT. Samples were washed 2 × 10 min at RT and mounted in Dako mounting medium. Fluorescence was observed using a Leica TCS SP2 confocal microscope.

### Fluorescence Quantification

Immunohistochemical analysis of DILP2 and DILP5 protein levels within the IPCs in whole mount brain of larva was performed as previously described (Géminard et al., 2009). Briefly, confocal images were obtained using a 40× objective using a 1 μm step size. Mean DILP fluorescence intensity in the IPCs was quantified from confocal z stack images using FIJI software (ImageJ 1.47k). A region adjacent to the IPCs served as background and was subtracted from the mean DILP fluorescence in the IPCs. To compare the different genotypes and feeding conditions, DILP values of either starved animals or control genotype that were not incubated in a medium containing leucine (Dilp2 > +) served as a reference (immunofluorescence = 1).

### Hemolymph Sugar Measurement

Hemolymph sugar measurements were performed as previously described (Géminard et al., 2009, Tennessen et al., 2014). L1 larvae were collected 24 hr after egg laying (4 hr egg collections) and reared at a density of 30 larvae/tube in standard corn/yeast medium at 25°C until they reach the L3 feeding stage. L3 Larvae were starved for 24 hr in starvation medium and transferred on starvation medium supplemented with 0.2% leucine. After 6 hr, 2 μL (approximately ten larvae) hemolymph of eight groups per genotype were collected. The hemolymph was diluted (1:10) in trehalase buffer (137 mM NaCl, 2.7 mM KCl, 5 mM Tris [pH 6.6]) and heated for 5 min at 70°C to inactivate the endogenous Trehalase. Trehalose was converted into glucose after incubation with porcine Trehalase (Sigma; T8778) at 37°C for 24 hr. The total amount of glucose was measured using the Glucose Hexokinase Assay kit (Sigma; GAHK20). The concentration of glucose was determined using a SPECTROstar (BMG LABTECH) plate reader at 450 nm.

### qRT-PCR

Larvae of various genotypes were reared until feeding third-instar stage. RNA from 80 brains/genotype was extracted using TRIzol (Invitrogen) and treated with RNase free DNase to eliminate genomic DNA. Total RNA (1 μg) was reverse transcribed using the iScript cDNA Synthesis kit (Bio-Rad). A standard protocol was used for real-time PCR (Applied Biosystems, Roche). PCR primers for *Dilp2* (atcccgtgattccacacaag and gcggttccgatatcgagtta) were designed for a region spanning from the second to the third exon. PCR primers for *Mnd*: ggacaatccctcatcgtttg and cctgatttgggtatcatcgtg.

### Weight Determination

Females were allowed to lay eggs during 4 hr and the resulting first-instar larvae were collected about 24 hr after the beginning of the egg laying. Larvae were reared at a density of 30 larvae/tube at 25°C in the poor medium containing: 5.1 g inactivated yeast powder, 12.44 g corn flour, 4.5 g sucrose, and 3 g Nipagin M (in ethanol) per liter (Géminard et al., 2009). Body weight determination was adapted from Géminard et al. (2009): for each genotype, body weight was determined 1 hr after hatching by weighing individual males with high precision weighting balance (Sartorius, R 160 P-^∗^F1).

### Statistical Analysis

All data were transferred to Prism 5.0d (Graphpad) for statistical analysis and tested for normal distribution using the D’Agostino and Pearson omnibus normality test. Normally distributed data were compared using the Student’s unpaired t test. Pairs of data that did not pass the normality test were analyzed using the Mann-Whitney test. Comparisons between sets of three or more normally distributed data were performed using the one-way ANOVA test followed by Bonferroni’s post hoc test. If the data were not normally distributed, they were compared using the Kruskal-Wallis test followed by Dunn’s post hoc test. Normally distributed data with two nominal variables were analyzed using the two-way ANOVA test followed by Bonferroni’s post hoc test.

## Author Contributions

G.M., A.B.Z., and Y.G. designed the overall strategy, including the conceptualization and the methodology. G.M., A.B.Z., and F.G. performed the experiments and collected the data. D.E.F. designed the anti-MND antibody. G.M., A.B.Z., and Y.G. wrote the paper with input from F.G. and D.E.F.

## Supplemental Information

Document S1. Supplemental Experimental Procedures and Figures S1–S4

Document S2. Article plus Supplemental Information
