# Supplementary material for: Direct Sensing of Nutrients via a LAT1-like Transporter in Drosophila Insulin-Producing Cells
Source: Cell Rep. 2016 Sep 27;17(1):137–48. doi: 10.1016/j.celrep.2016.08.093 (PMC5055474; doi:10.1016/j.celrep.2016.08.093)
Supplement: Document S1. Supplemental Experimental Procedures and Figures S1–S4 [file mmc1.pdf]

Cell Reports, Volume *17*

Supplemental Information

Direct Sensing of Nutrients via a LAT1-like Transporter in *Drosophila* Insulin-Producing Cells

Gérard Manière, Anna B. Ziegler, Flore Geillon, David E. Featherstone, and Yael Grosjean

Supplemental material and methods:

Western blot and dot blot:

Adult heads were homogenized in lysis buffer (50 mM Tris [pH 8], 150 mM NaCl, 1% Triton X-100 Complete Roche protease inhibitor). Starved and leucine-refed third-instar larvae were collected and washed in cold PBS and hemolymph extracted using a glass capillary. Samples were quantified using the Lowry method and denatured in 2X Laemmli buffer with 150 mM β-mercaptoethanol. For western blots, proteins were loaded onto gradient precast gel (4%-20% mini protean TGX, Biorad) and then transferred on nitrocellulose membrane (0.2 µm, Biorad). For dot blots, proteins (in a volume of 1 or 2 µL) were adsorbed on nitrocellulose membrane dried 1 h at room temperature. The membrane was probed with 1:1000 diluted monoclonal anti-FLAG antibody (clone M2, Sigma) and then with the appropriate secondary antibody coupled to horseradish peroxidase (1:10,000 Santa Cruz Biotechnology) in TBS with 3% non-fat milk, before being revealed by ECL (ECL Prime, Amersham). Image processing and analysis were done using Chemi Doc XRS + Imaging System (Bio-Rad) and the Image Lab software.

Enzyme immunoassay (EIA) for hemolymph Dilp:

The protocol was directly adapted from Bai et al. 2012. One µL of hemolymph was diluted in 50 µL of PBS and incubated overnight in cells of a 96-well EIA at room temperature. Following incubation, cells were cleared of hemolymph, and bound material in the plate was blocked for 2 h with EIA buffer (10 mM Na_2HPO_4, 3 mM NaH_2PO_4, 150 mM NaCl, 1 mM EDTA, 0.1% thimerosal) and 1% BSA. Blocked samples were washed three times with PBS-Tween 0.2%. Except for the blank well, samples were treated with 100 µL of anti-DILP2 or anti-DILP5 antibody (provided by P. Leopold laboratory; Geminard et al., 2009) at 1:2500 dilution, incubated 2 h at room temperature, washed three times with PBS-Tween, and treated with HRP-conjugated secondary antibody (1:2500, Santa Cruz Biotechnology). In the final step, the plate was washed and treated with TMB solution (liquid substrate system for ELISA, Sigma) to provide colorimetric quantification. This reaction was stopped by 100 µL 1 M phosphoric acid, and absorbance was recorded at 450 nm using Spectrostar^{Nano} spectrophotometer (BMG LABTECH).

Supplemental figure legends:

Supplemental Figure 1: Anti-MND localization (magenta) in the fat body using *OK376*-Gal4 driver (Bloomington stock Nr. 6487, Kunte et al., 2006). (A) Co-localization with UAS-mCD8::GFP (plasma membrane, green), (B) with UAS-KDEL::GFP (endoplasmic reticulum, green). Arrows indicate co-localizations.

Supplemental Figure 2: Quantified DILP2 (A) and DILP5 (B) immunofluorescence intensities in IPCs of isolated brains incubated in Schneider´s medium supplemented with 20 mM leucine do not increase between the control genotype (grey) and animals in which the TOR pathway has been impaired by either RNAi-mediated down-regulation of *Raptor,* a member of mTORC1 (light blue), or the expression of a dominant negative and thereby inactive form of TOR (dark blue).

Supplemental Figure 3: Western blot (A) and dot blot (B) detection of FLAG-tagged DILP2 (HA-FLAG-tagged DILP2, Ilp2HF, Park *et al.* 2014), using an anti-FLAG antibody. Head protein lysate obtained from flies overexpressing Ilp2HF in eyes is used as a positive control. While western blot conditions are suitable for specific detection of Ilp2HF in 25 µg and 50 µg of adult head protein lysate, the protein cannot be detected in 150 µg of hemolymph proteins collected from starved larvae overexpressing tagged DILP2 in insulin producing cells, fed or not for 6 h with starvation medium supplemented with 20 mM leucine. Unlike in western blots, Ilp2HF cannot be specifically detected by dot blot performed with 0.5 to 4 µg of adult head protein lysate.

Supplemental Figure 4: Larval hemolymph DILP measurement by Enzyme immunoassay (EIA; following the protocol described in Bai et al. 2012). (A) Hemolymph are collected from starved larvae, fed or not for 6 h with starvation medium supplemented with 20 mM leucine. DILP2 and DILP5 circulating levels are indistinguishable either between the control genotype (Dilp2>+) and when *Mnd* is under-expressed in IPCs (Dilp2>Mnd^{dsRNA}), or depending on the feeding conditions. (B) Larval circulating levels of DILP2 and DILP5 in control (Dilp2>+) are indistinguishable when starved larvae are fed for 15 min to 2 h with starvation medium supplemented with 20 mM leucine.

Supplemental references:

Kunte, A.S., Matthews, K.A, and Rawson, R.B. (2006). Fatty acid auxotrophy in *Drosophila* larvae lacking SREBP. Cell Metab *3*, 439–448.

Park, S., Alfa, R.W., Topper, S.M., Kim, G.E., Kockel, L., and Kim, S.K. (2014) A genetic strategy to measure circulating Drosophila insulin reveals genes regulating insulin production and secretion. PLoS Genet *10*, e1004555.

Suppl Fig 1

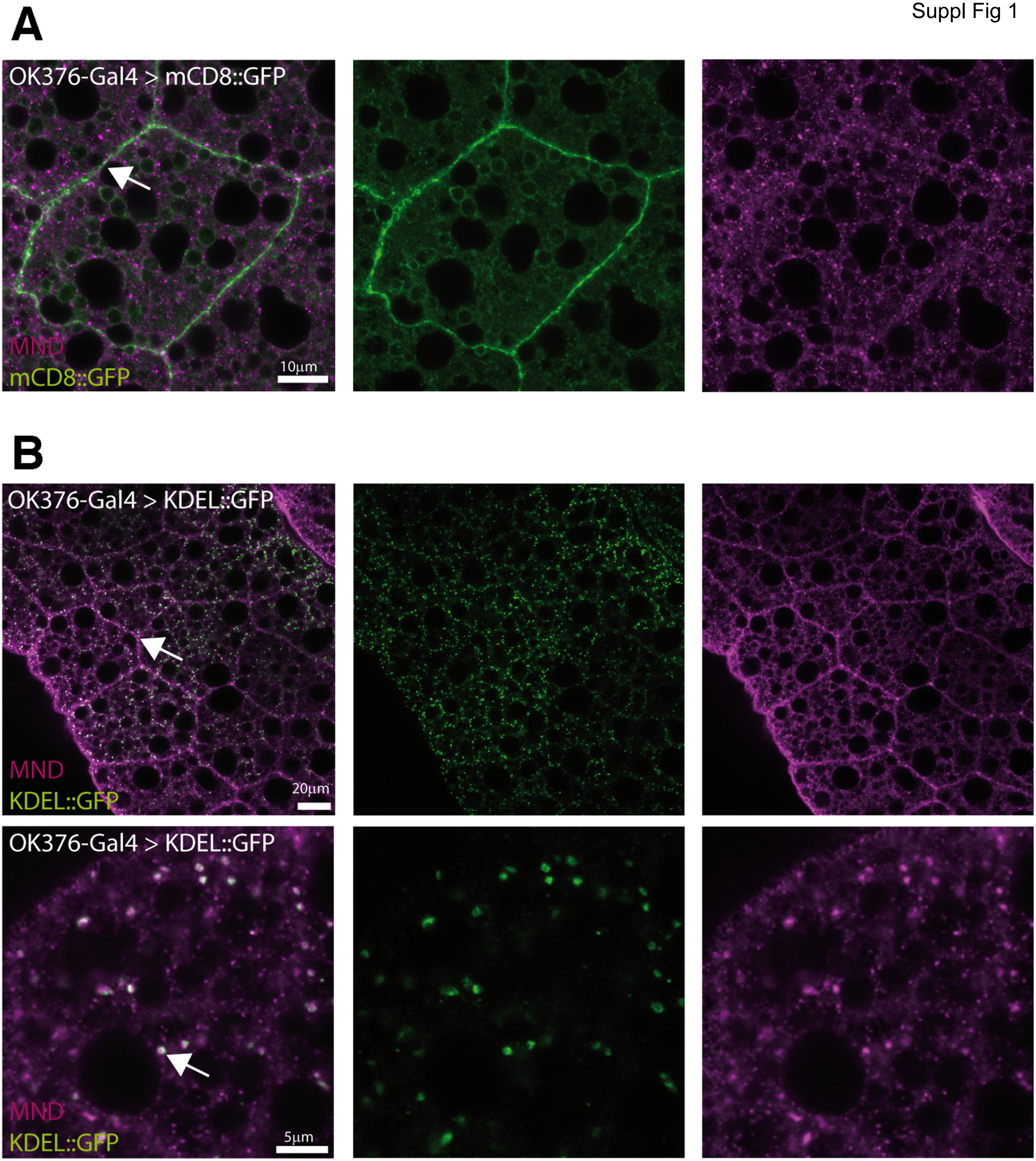

A
OK376-Gal4 > mCD8::GFP
MND
mCD8::GFP
10µm
B
OK376-Gal4 > KDEL::GFP
MND
KDEL::GFP
20µm
OK376-Gal4 > KDEL::GFP
MND
KDEL::GFP
5µm

Suppl Fig 2

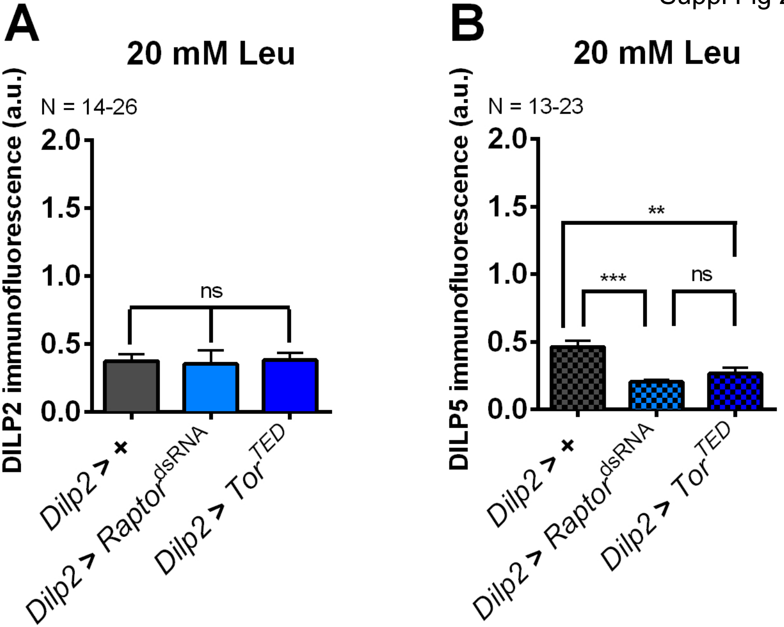
A
20 mM Leu
N = 14-26
DILP2 immunofluorescence (a.u.)
2.0
1.5
1.0
0.5
0.0
ns
Dilp2 > +
Dilp2 > Raptor dsRNA
Dilp2 > Tor TED
B
20 mM Leu
N = 13-23
DILP5 immunofluorescence (a.u.)
2.0
1.5
1.0
0.5
0.0
**
***
ns
Dilp2 > +
Dilp2 > Raptor dsRNA
Dilp2 > Tor TED

Suppl Fig 3

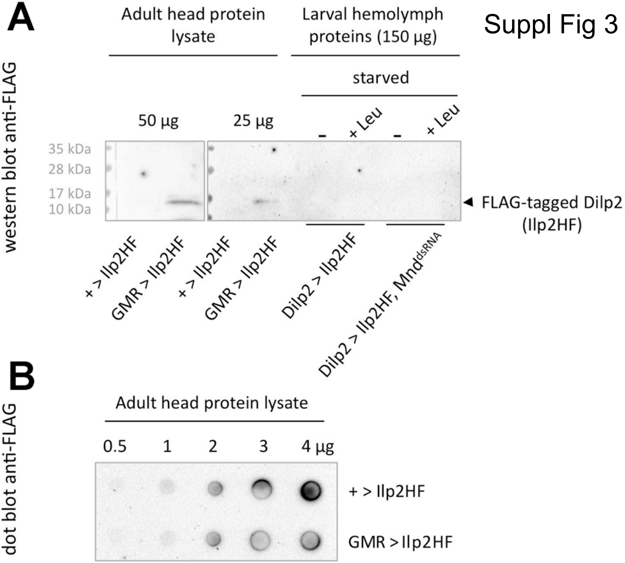
A
Adult head protein lysate
Larval hemolymph proteins (150 µg)
starved
50 µg
25 µg
–
+ Leu
–
+ Leu
western blot anti-FLAG
35 kDa
28 kDa
17 kDa
10 kDa
FLAG-tagged Dilp2 (Ilp2HF)
+ > Ilp2HF
GMR > Ilp2HF
+ > Ilp2HF
GMR > Ilp2HF
Dilp2 > Ilp2HF
Dilp2 > Ilp2HF, Mnd^dsRNA
B
Adult head protein lysate
dot blot anti-FLAG
0.5
1
2
3
4 µg
+ > Ilp2HF
GMR > Ilp2HF

Suppl Fig 4

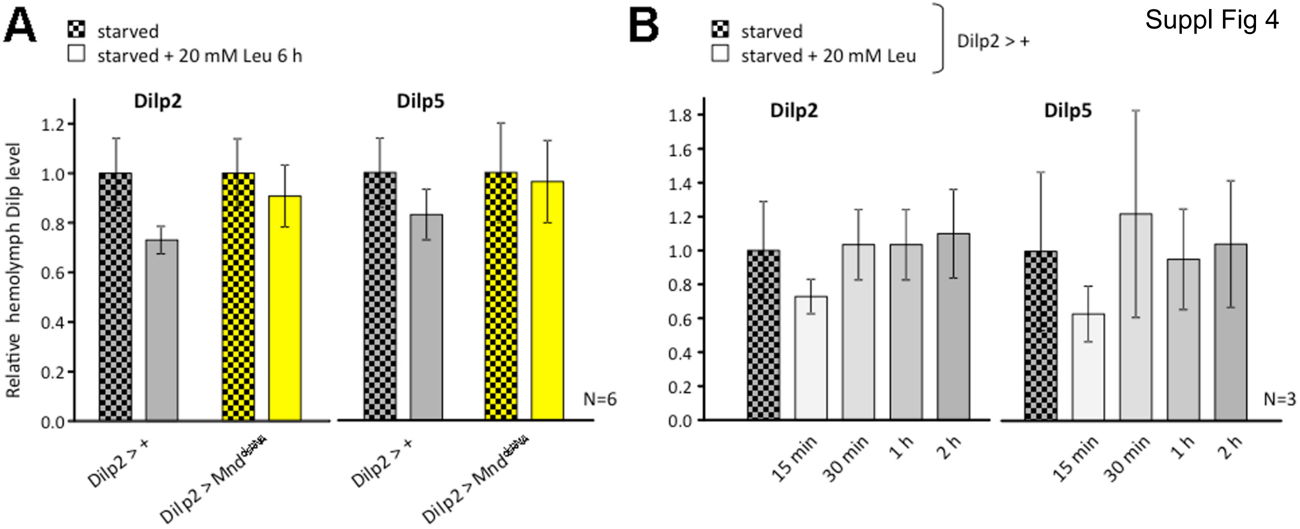
A
starved
starved + 20 mM Leu 6 h
Dilp2
Dilp5
Relative hemolymph Dilp level
1.2
1.0
0.8
0.6
0.4
0.2
0.0
N=6
Dilp2 > +
Dilp2 > Mnd
Dilp2 > +
Dilp2 > Mnd
B
starved
starved + 20 mM Leu
Dilp2 > +
Dilp2
Dilp5
1.8
1.6
1.4
1.2
1.0
0.8
0.6
0.4
0.2
0.0
N=3
15 min
30 min
1 h
2 h
15 min
30 min
1 h
2 h
